# Supplementary material for: GWAS meta-analysis reveals key risk loci in essential tremor pathogenesis
Source: Commun Biol. 2024 Apr 26;7:504. doi: 10.1038/s42003-024-06207-4 (PMC11053069; doi:10.1038/s42003-024-06207-4)
Supplement: Supplementary file 2 — Supplementary Information [file 42003_2024_6207_MOESM2_ESM.docx]

# **Supplementary Information**

# **CA3 plays a role in essential tremor pathogenesis: A GWAS meta-analysis**

Astros Th. Skuladottir, Lilja Stefansdottir, Gisli H. Halldorsson, Olafur A. Stefansson, Anna Bjornsdottir, Palmi Jonsson, Vala Palmadottir, Thorgeir E. Thorgeirsson, G. Bragi Walters, Rosa S. Gisladottir, Gyda Bjornsdottir, Gudrun A. Jonsdottir, Patrick Sulem, Daniel F. Gudbjartsson, Kirk U. Knowlton, David A. Jones, Aigar Ottas, Estonian Biobank, Ole B. Pedersen, Maria Didriksen, Søren Brunak, Karina Banasik, Thomas Folkmann Hansen, Christian Erikstrup, DBDS Genomic Consortium, Jan Haavik, Ole A. Andreassen, David Rye, Jannicke Igland, Sisse Rye Ostrowski, Lili A. Milani, Lincoln D. Nadauld, Hreinn Stefansson, and Kari Stefansson

**Contents**

[**Supplementary Figure 1. Regional plots of the loci associating with ET.** 2](#_Toc144388077)

[**Supplementary Figure 2. Manhattan plot of individual dataset.** 4](#_Toc144388078)

[**Supplementary Figure 3. Forest plot of effects.** 6](#_Toc144388079)


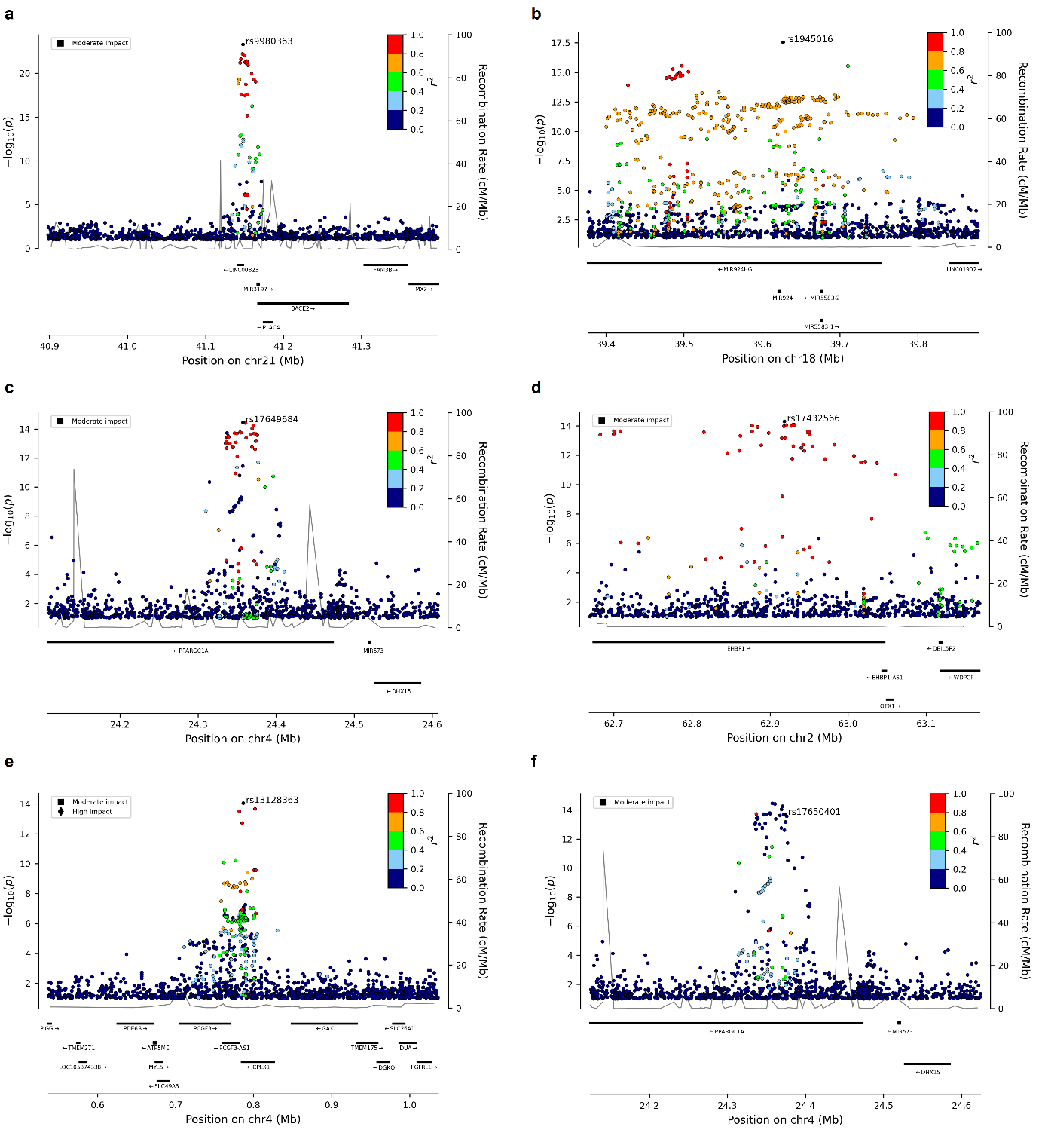


#
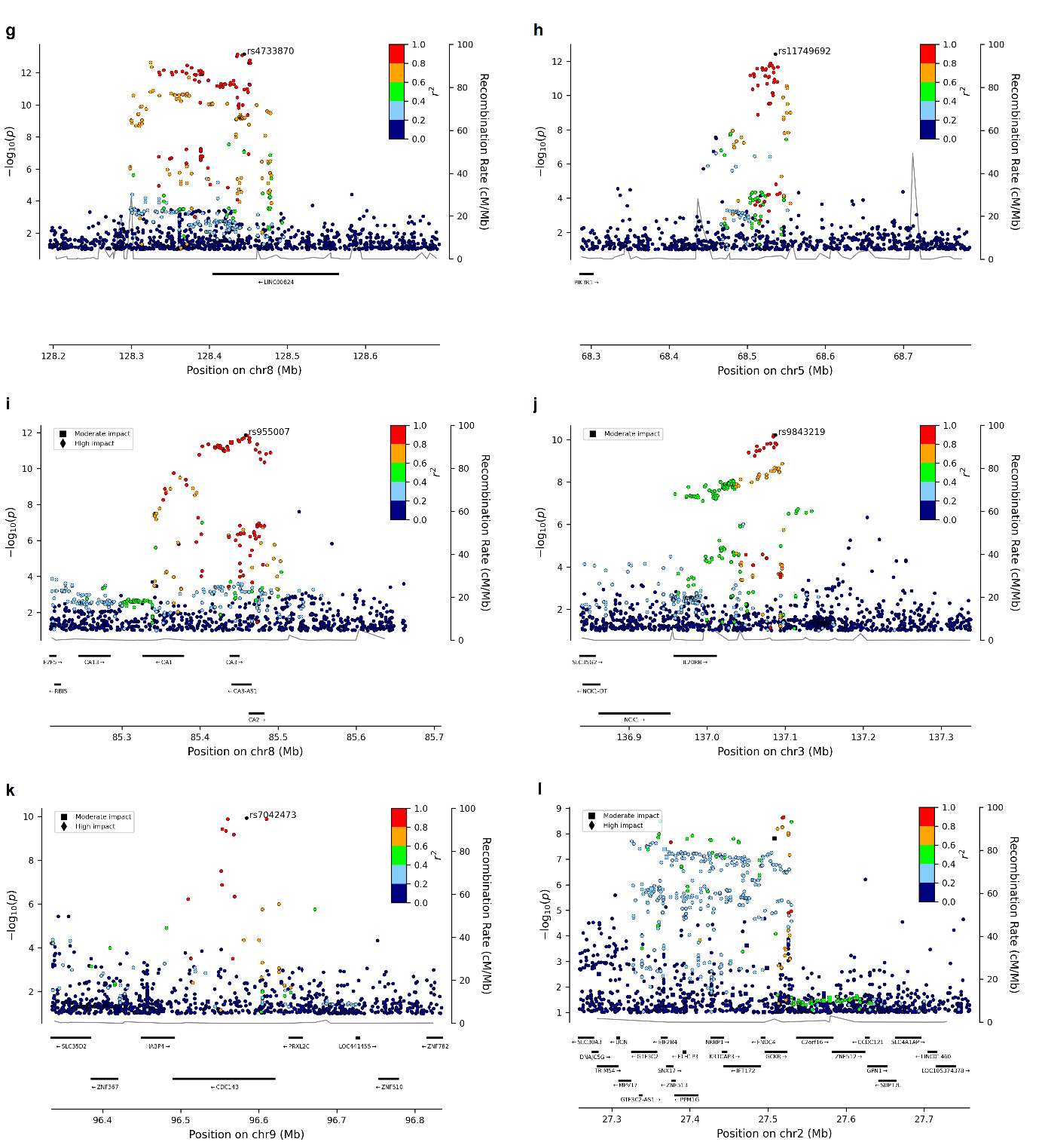
 **Supplementary Figure 1. Regional plots of the loci associating with ET.**

Variants are colored by the degree of correlation (*r*^2^) with the lead variant, which is colored black. Functional variants have a squared (moderate impact) or a diamond shape (high impact). The -log_10_*P*-values on the left y-axis (two-sided logistic regression) are plotted for each variant against their chromosomal position (x axis). The right y-axis shows calculated recombination rates based on the Icelandic data at the chromosomal location, plotted as solid black lines. *P*-values are two-sided and derived from a likelihood-ratio test.


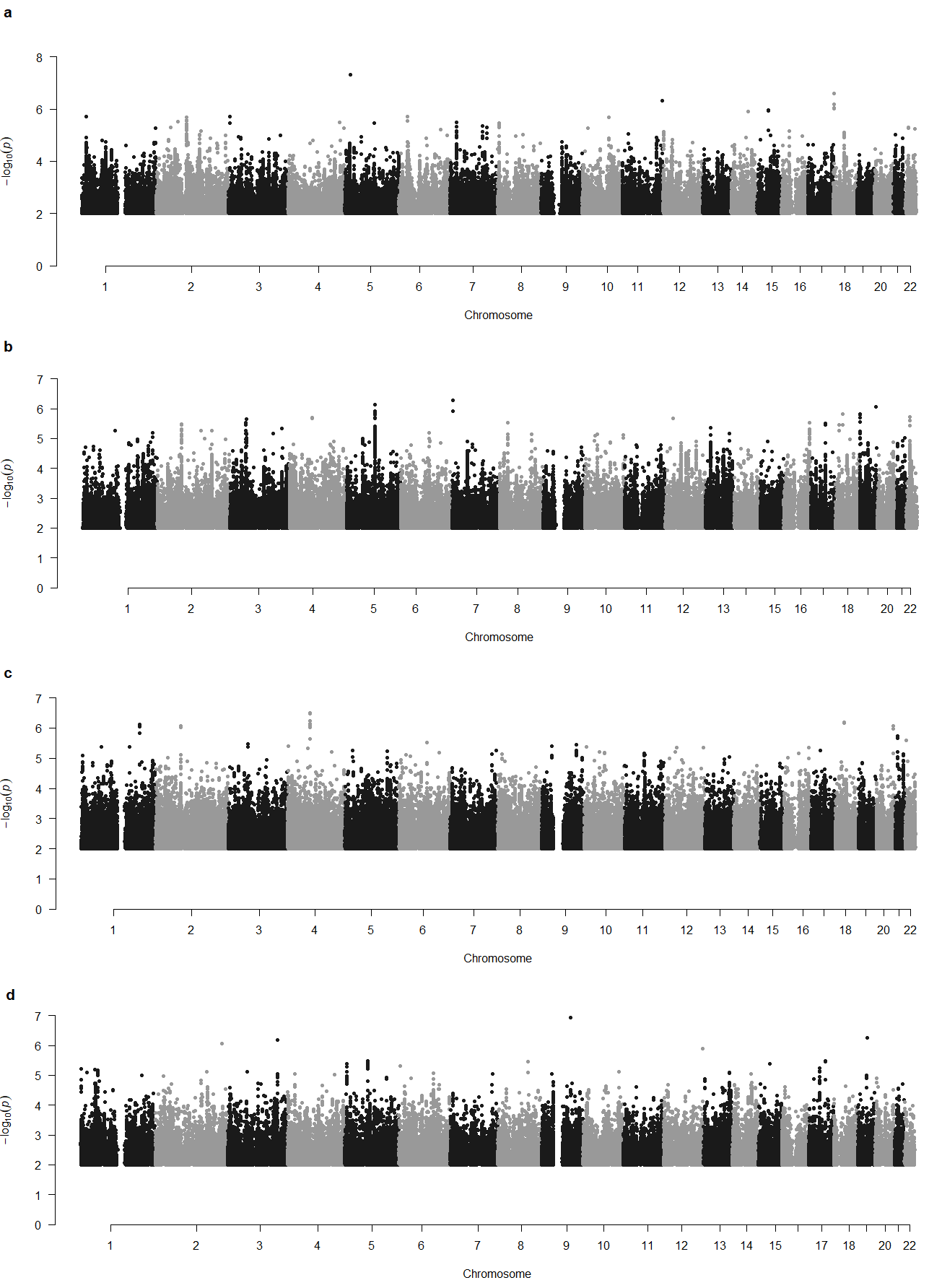


#
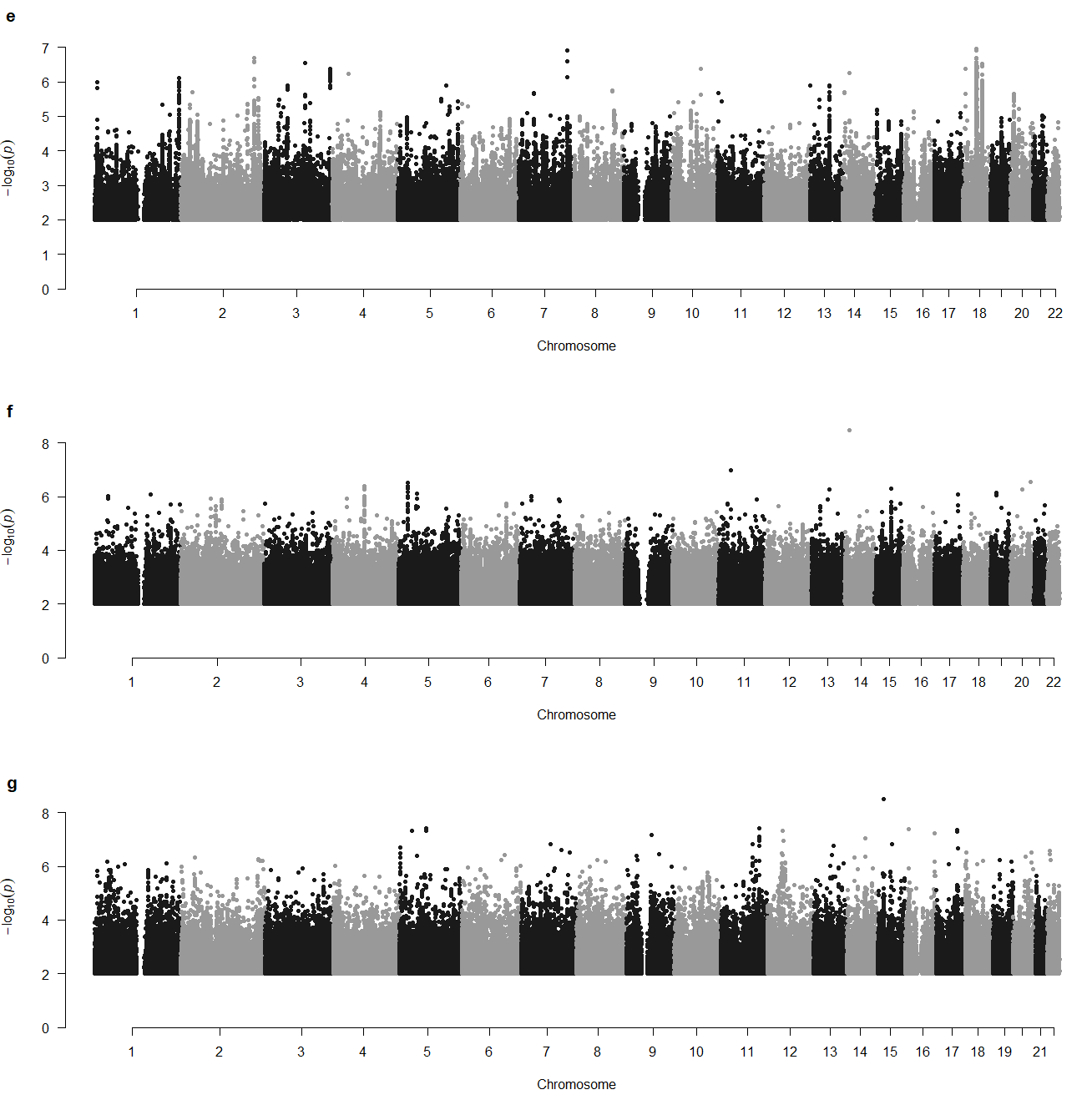
 **Supplementary Figure 2. Manhattan plot of individual dataset.**

Manhattan plots showing GWAS results under an additive model for a) Iceland, b) Denmark, c) Estonia, d) Norway, e) The UK, f) The US – INTMT, and g) The US – EMORY. The -log_10_*P*-values (y-axis) are plotted for each variant against their chromosomal position (x-axis). *P*-values are two-sided and derived from a likelihood-ratio test.

#
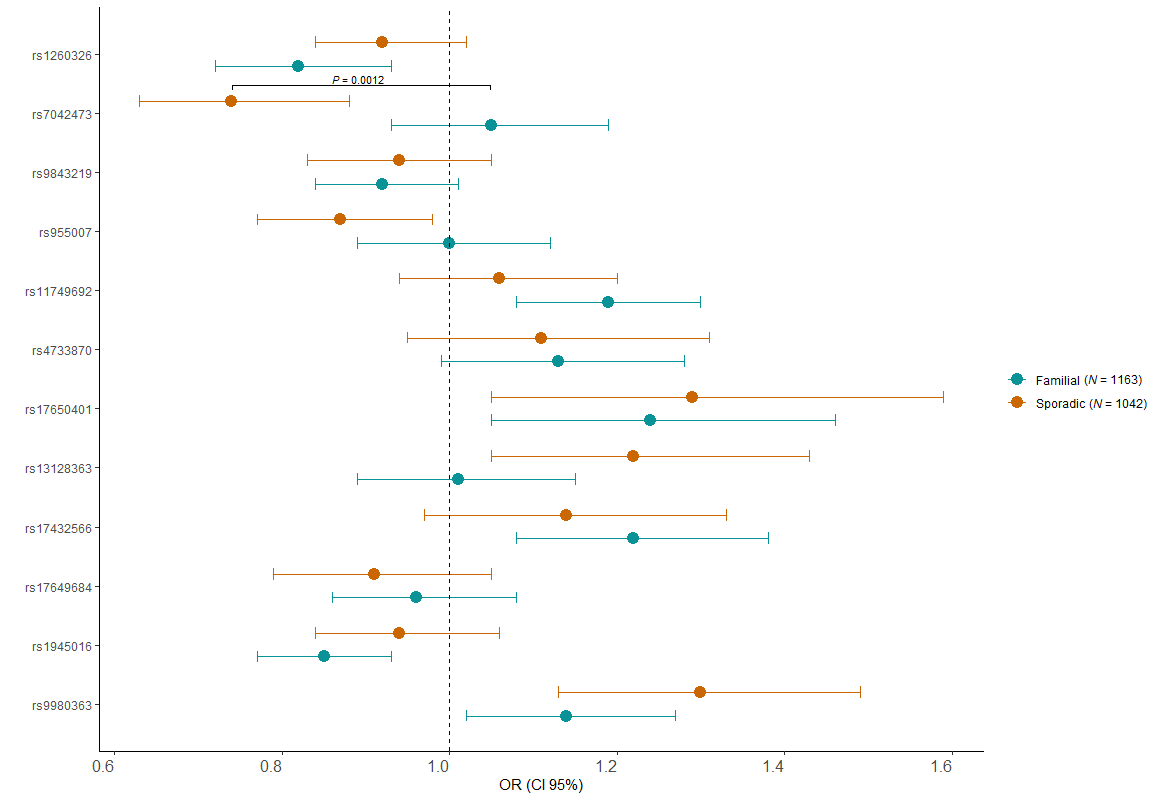
 **Supplementary Figure 3. Forest plot of effects.**

Forest plot showing the comparison of direction of effects (OR) between familial and sporadic cases for the 12 variants associated with ET. Familial cases were clustered based on having any first or second degree relative. The error bars represent the 95% confidence interval.
