# Supplementary material for: GWAS meta-analysis reveals key risk loci in essential tremor pathogenesis
Source: Commun Biol. 2024 Apr 26;7:504. doi: 10.1038/s42003-024-06207-4 (PMC11053069; doi:10.1038/s42003-024-06207-4)
Supplement: Supplementary file 3 — Description of Additional Supplementary Files [file 42003_2024_6207_MOESM3_ESM.docx]

**Description of Additional Supplementary Files**

**File name:** Supplementary Data 1

**Description:** Demographics of the essential tremor datasets.

**File name:** Supplementary Data 2

**Description:** Wighted genome-wide significance thresholds.

**File name:** Supplementary Data 3

**Description:** Genome-wide associations of ET meta-analysis

**File name:** Supplementary Data 4

**Description:** Conditional analysis

**File name:** Supplementary Data 5

**Description:** Previously reported associations with ET and correlated top signals in the ET meta-analysis

**File name:** Supplementary Data 6

**Description:** Coding variants

**File name:** Supplementary Data 7

**Description:** cis-eQTL

**File name:** Supplementary Data 8

**Description:** CA3 pQTLs

**File name:** Supplementary Data 9

**Description:** GCKR pQTLs

**File name:** Supplementary Data 10

**Description:** Gene-set analysis in FUMA

**File name:** Supplementary Data 11

**Description:** Sex-specific model

**File name:** Supplementary Data 12

**Description:** Estimation of genetic variance explained

**File name:** Supplementary Data 13

**Description:** Genetic correlation as estimated with LD score regression between essential tremor and 1152 GWASs

**File name:** Supplementary Data 14

**Description:** Association analysis covariates

**File name:** Supplementary Data 15

**Description:** Data sources for expression quantitative trait loci (eQTL) results presented in Supplementary Data 7
