# Supplementary material for: GWAS meta-analysis reveals key risk loci in essential tremor pathogenesis
Source: Commun Biol. 2024 Apr 26;7:504. doi: 10.1038/s42003-024-06207-4 (PMC11053069; doi:10.1038/s42003-024-06207-4)
Supplement: Supplementary file 5 — Reporting Summary [file 42003_2024_6207_MOESM5_ESM.pdf]

nature portfolio

Corresponding author(s): Astros Th. Skuladottir
Kari Stefansson

Last updated by author(s): Mar 20, 2024

Reporting Summary

Nature Portfolio wishes to improve the reproducibility of the work that we publish. This form provides structure for consistency and transparency in reporting. For further information on Nature Portfolio policies, see our Editorial Policies and the Editorial Policy Checklist.

Statistics

For all statistical analyses, confirm that the following items are present in the figure legend, table legend, main text, or Methods section.

n/a	Confirmed	
☐	☒	The exact sample size (*n*) for each experimental group/condition, given as a discrete number and unit of measurement
☐	☒	A statement on whether measurements were taken from distinct samples or whether the same sample was measured repeatedly
☐	☒	The statistical test(s) used AND whether they are one- or two-sided<br>*Only common tests should be described solely by name; describe more complex techniques in the Methods section.*
☐	☒	A description of all covariates tested
☐	☒	A description of any assumptions or corrections, such as tests of normality and adjustment for multiple comparisons
☐	☒	A full description of the statistical parameters including central tendency (e.g. means) or other basic estimates (e.g. regression coefficient) AND variation (e.g. standard deviation) or associated estimates of uncertainty (e.g. confidence intervals)
☐	☒	For null hypothesis testing, the test statistic (e.g. *F*, *t*, *r*) with confidence intervals, effect sizes, degrees of freedom and *P* value noted<br>*Give P values as exact values whenever suitable.*
☒	☐	For Bayesian analysis, information on the choice of priors and Markov chain Monte Carlo settings
☒	☐	For hierarchical and complex designs, identification of the appropriate level for tests and full reporting of outcomes
☒	☐	Estimates of effect sizes (e.g. Cohen's *d*, Pearson's *r*), indicating how they were calculated

Our web collection on statistics for biologists contains articles on many of the points above.

Software and code

Policy information about availability of computer code

Data collection

No software was used for data collection

Data analysis

We used the following publicly available software to analyze the data:

GraphTyper (v2.0-beta, GNU GPLv3 license) at https://github.com/DecodeGenetics/graphtyper

Svimmer (v0.1, GNU GPLv3 license), the structural variant merging software at https://github.com/DecodeGenetics/svimmer

SHAPEIT4 (v4.2.2) at https://odelaneau.github.io/shapeit4/

Eagle2 (v2.4.1) at http://www.hsph.harvard.edu/alkes-price/software/

Beagle (v5.4) at https://faculty.washington.edu/browning/beagle/beagle.html

GCTA (v1.93.3beta2) at https://yanglab.westlake.edu.cn/software/gcta/#Overview

STAR (v2.5.3) at http://star.mit.edu/

Kallisto at https://pachterlab.github.io/kallisto/

LeafCutter at https://davidaknowles.github.io/leafcutter/

LD score regression (first release) at https://github.com/bulik/ldsc

qqman package (v0.1.6) at https://github.com/stephenturner/qqman

Axiom genotyping algorithm (v1) at https://www.thermofisher.com/is/en/home.html

FUMA at https://fuma.ctglab.nl/

We used version 3.6.3 of R and version 1.2.5042 of RStudio for statistical analyses and graphs and figures.

For manuscripts utilizing custom algorithms or software that are central to the research but not yet described in published literature, software must be made available to editors and reviewers. We strongly encourage code deposition in a community repository (e.g. GitHub). See the Nature Portfolio guidelines for submitting code & software for further information.

Data

Policy information about availability of data

All manuscripts must include a data availability statement. This statement should provide the following information, where applicable:

- Accession codes, unique identifiers, or web links for publicly available datasets
- A description of any restrictions on data availability
- For clinical datasets or third party data, please ensure that the statement adheres to our policy

The GWAS summary statistics for the ET meta-analysis will be made available at https://www.decode.com/summarydata/. Other data generated or analyzed in this study are included in the article and Supplementary data and information.

Research involving human participants, their data, or biological material

Policy information about studies with human participants or human data. See also policy information about sex, gender (identity/presentation), and sexual orientation and race, ethnicity and racism.

Reporting on sex and gender	In the manuscript we only use "sex". We did not have gender information. We applied sex-specific models to the Icelandic, Danish, Norwegian, UK, and US-INTMT datasets for the genome-wide significant variants and showed no significant difference between the sexes. We also report the sex ratio.
Reporting on race, ethnicity, or other socially relevant groupings	One of the limitations of the study is the lack of ethnic diversity. Unfortunately, we did not have access to essential tremor cases of other than European background.
Population characteristics	In this study, we used subjects diagnosed with essential tremor and controls of European descent that have given an informed consent to participate in large genetic studies. Of the total cases, 52.9% were females and 47.1% males and the average age at diagnosis was 62 years.
Recruitment	The data used in the GWAS meta-analysis were collected through studies approved by ethics committees governing each dataset and written informed consent was obtained from all participants. In each dataset, we searched records from hospitals, private practice, and other health records for ICD-10 code G25.0 and ICD-9 code 333.1.
Ethics oversight	Iceland: The data in this study was approved by the National Bioethics Committee (VSN-17-142-V5; VSNb2017060004/03.01) following review by the Icelandic Data Protection Authority.<br><br>Denmark: Data analysis was performed under the "Developing the basis for personalized medicine in degenerative and episodic brain disorders" protocol, approved by the National Committee on Health Research Ethics (H-21058057). The Danish Data Protection Agency (P-2019-99) and the National Committee on Health Research Ethics (NVK-1700407) approved the studies under which data on DBDS participants were obtained.<br><br>Estonia: Analysis of individual level data from the EstBB was carried out under ethical approval 1.1-12/624 from the Estonian Committee on Bioethics and Human Research (Estonian Ministry of Social Affairs), using data according to release application [6-7/GI/29 977] from the Estonian Biobank.<br><br>Norway: The HUSKment study is approved by the Regional Committee for Medical Research Ethics Western Norway, reference 2018/915.<br><br>The UK: The North West Research Ethics Committee reviewed and approved UK Biobank's scientific protocol and operational procedures (REC Reference Number: 06/MRE08/65). This study was conducted using the UK Biobank resource under application number 42256.<br><br>The US-INTMT: The Intermountain Healthcare Institutional Review Board approved this study.<br><br>The US-EMORY: The study was approved by the Emory Institutional Review Board.

Note that full information on the approval of the study protocol must also be provided in the manuscript.

Field-specific reporting

Please select the one below that is the best fit for your research. If you are not sure, read the appropriate sections before making your selection.

☒ Life sciences ☐ Behavioural & social sciences ☐ Ecological, evolutionary & environmental sciences

For a reference copy of the document with all sections, see nature.com/documents/nr-reporting-summary-flat.pdf

Life sciences study design

All studies must disclose on these points even when the disclosure is negative.

Sample size	The sample size was determined by combining all available subjects that had been diagnosed with essential tremor in the all datasets available.
Data exclusions	We excluded variants with imputation information below 0.8 and MAF below 0.01% for quality reasons.
Replication	Our study is a meta-analysis of GWAS results for essential tremor where we combined cases and controls from available datasets. Thus, there was no direct replication involved. However, we studied associations of five previously reported variants associating with essential tremor. We studied these associations in a larger sample size. We found support for four of them.
Randomization	No randomizations were used in the study as it is based on a GWAS meta-analysis. The data collected was based on individuals with ICD-10 codes G25.0 in hospital registries. Participation in studies or surveys, particularly genetic studies, is biased toward those wanting to participate (https://www.biorxiv.org/content/10.1101/2022.02.11.480067v1). Randomizing an already biased sample will have a limited effect.
Blinding	This is an observational association study and no blinding was required.

Reporting for specific materials, systems and methods

We require information from authors about some types of materials, experimental systems and methods used in many studies. Here, indicate whether each material, system or method listed is relevant to your study. If you are not sure if a list item applies to your research, read the appropriate section before selecting a response.

Materials & experimental systems

n/a	Involved in the study	
☒	☐	Antibodies
☒	☐	Eukaryotic cell lines
☒	☐	Palaeontology and archaeology
☒	☐	Animals and other organisms
☒	☐	Clinical data
☒	☐	Dual use research of concern
☒	☐	Plants

Methods

n/a	Involved in the study	
☒	☐	ChIP-seq
☒	☐	Flow cytometry
☒	☐	MRI-based neuroimaging

Plants

Seed stocks	N/A
Novel plant genotypes	N/A
Authentication	N/A
